# Genome-wide DNA methylation analysis reveals hypomethylation in the low-CpG promoter regions in lymphoblastoid cell lines

**DOI:** 10.1186/s40246-017-0106-6

**Published:** 2017-05-12

**Authors:** Itsuki Taniguchi, Chihiro Iwaya, Keizo Ohnaka, Hiroki Shibata, Ken Yamamoto

**Affiliations:** 1 Division of Genomics, Medical Institute of Bioregulation, Kyushu University, 3-1-1 Maidashi, Higashi-ku, Fukuoka, 812-8582 Japan; 2 Department of Geriatric Medicine, Graduate School of Medical Sciences, Kyushu University, 3-1-1 Maidashi, Higashi-ku, Fukuoka, 812-8582 Japan; 3 Department of Medical Biochemistry, Kurume University School of Medicine, 67 Asahi-machi, Kurume, Fukuoka 830-0011 Japan

**Keywords:** DNA methylation, Lymphoblastoid cell lines, Epigenome-wide analysis, Epigenetic epidemiology, Human methylation array

## Abstract

**Background:**

Epidemiological studies of DNA methylation profiles may uncover the molecular mechanisms through which genetic and environmental factors contribute to the risk of multifactorial diseases. There are two types of commonly used DNA bioresources, peripheral blood cells (PBCs) and EBV-transformed lymphoblastoid cell lines (LCLs), which are available for genetic epidemiological studies. Therefore, to extend our knowledge of the difference in DNA methylation status between LCLs and PBCs is important in human population studies that use these DNA sources to elucidate the epigenetic risks for multifactorial diseases. We analyzed the methylation status of the autosomes for 192 and 92 DNA samples that were obtained from PBCs and LCLs, respectively, using a human methylation 450 K array. After excluding SNP-associated methylation sites and low-call sites, 400,240 sites were subjected to analysis using a generalized linear model with cell type, sex, and age as the independent variables.

**Results:**

We found that the large proportion of sites showed lower methylation levels in LCLs compared with PBCs, which is consistent with previous reports. We also found that significantly different methylation sites tend to be located on the outside of the CpG island and in a region relatively far from the transcription start site. Additionally, we observed that the methylation change of the sites in the low-CpG promoter region was remarkable. Finally, it was shown that the correlation between the chronological age and ageing-associated methylation sites in *ELOVL2* and *FHL2* in the LCLs was weaker than that in the PBCs.

**Conclusions:**

The methylation levels of highly methylated sites of the low-CpG-density promoters in PBCs decreased in the LCLs, suggesting that the methylation sites located in low-CpG-density promoters could be sensitive to demethylation in LCLs. Despite being generated from a single cell type, LCLs may not always be a proxy for DNA from PBCs in studies of epigenome-wide analysis attempting to elucidate the role of epigenetic change in disease risks.

**Electronic supplementary material:**

The online version of this article (doi:10.1186/s40246-017-0106-6) contains supplementary material, which is available to authorized users.

## Background

The DNA obtained from EBV-transformed immortalized lymphoblastoid cell lines (LCLs) and peripheral blood cells (PBCs) are commonly used in medical genetic studies. LCLs can be generated from both healthy individuals and patients and supply an unlimited source of genomic DNA. Additionally, LCLs and PBCs have been successfully used for gene expression analyses [1].

DNA methylation is one of the important epigenetic mechanisms regulating gene expression. In addition to sequence variants, it is increasingly accepted that this DNA modification may be implicated in the susceptibility of various multifactorial diseases [2–4]. Recent developments in technology for human genome analysis have enabled us to identify disease-related DNA methylation changes at the genome-wide level. Because it is essential to use relatively large samples in searching for genes that are susceptible to multifactorial diseases, the DNA sources are limited to LCLs, PBCs, and saliva. However, it is known that DNA methylation status varies between cell types [5]. Therefore, to extend our knowledge of the difference in DNA methylation status between LCLs and PBCs is important in human population studies that use these DNA sources to elucidate the epigenetic risks for multifactorial diseases.

To this end, we designed experiments to compare the DNA methylation status between LCLs and PBCs at an epigenome-wide level using approximately 400,000 methylation data sites from 92 LCL and 192 PBC samples obtained using the Human Methylation 450 K array. We analyzed global differences in methylation profiles and the degree of difference in methylation level of each site in terms of location (inside or outside the CpG island, the distance from transcription start site and promoter type) between LCLs and PBCs. Additionally, the association strength of methylation levels at the ageing-related methylation sites in *FHL2* and *ELOVL2* with chronological age was compared between LCLs and PBCs.

## Methods

### Subjects

EBV-transformed LCLs derived from 92 healthy Japanese subjects were provided by the Riken Bioresource Center Cell Bank [6]. PBCs were obtained from 192 participants of a baseline survey of the general population from a Fukuoka-based cohort study [7, 8]. This study was performed in accordance with the principles of the Declaration of Helsinki and was approved by the Institutional Review Board at Kyushu University.

### DNA methylation chip assay

Genomic DNA was bisulfite-treated using the EZ-96 DNA Methylation Kit (Zymo Research Corporation, Orange, CA), which combines bisulfite conversion and DNA cleanup in a 96-well plate. Genome-wide DNA methylation profiles were obtained using the Illumina HumanMethylation450 BeadChip (Illumina, San Diego, CA) according to the manufacturer’s instructions. The GenomeStudio V2011.1 (Methylation Module version 1.9.0) was employed to determine the beta values that reflected the estimated methylation level for each CpG site. The beta value was calculated as: Max(signal for methylation, 0)/[Max(signal for methylation, 0) + Max(signal for unmethylation, 0) + 100]. Using this metric, the DNA methylation level was represented by a number between 0 (no methylation) and 1 (complete methylation). The signal intensities were normalized to the internal controls and background prior to beta value calculation.

### Selection and classification of DNA methylation sites

Among 473,864 methylation sites on the autosomes, 1305 sites showing low calls (<0.95) were removed for further analyses. To eliminate SNP-associated methylation sites, we screened the nearest SNP for each methylation site using the dbSNP135 database (SNPs categorized in weight = 1 group, http://www.ncbi.nlm.nih.gov/SNP/). We found 72,318 sites in which SNPs were located on the C or G site. Additionally, one methylation site demonstrated an outlier value. After removing these sites; 400,240 methylation sites on the array were available for further analyses. Based on the CpG Islands (CGI) track of the UCSC table browser of the UCSC Genome Bioinformatics database (http://genome.ucsc.edu/index.html), the 400,240 sites on autosomes were classified into two groups, CGI-sites (135,674 sites, inside of CGI) or non-CGI-sites (264,566 sites, outside the CGI). Among the non-CGI sites, 95,625 sites were located near CGI (±2,000 bases) that were classified in a shore group. The distance between the methylation site and the nearest transcription start site (TSS) was calculated using the NCBI RefSeq database. The physical positions on the human genome were based on the Genome Reference Consortium Human Build 37 (GRCh37, http://www.ncbi.nlm.nih.gov/assembly/). Of 400,240 probes, 159,688 demonstrated a TSS between −500 bases and +2,000 bases; among these, 85,700 sites could be classified into high-CpG-density promoters (HCP), intermediate-CpG-density promoters (ICP) and low-CpG-density promoters (LCP), as reported by Mikkelsen et al. [9] (69,836, 10,719, and 5145 in HCP, ICP, and LCP, respectively).

### Statistical analysis

To evaluate the difference in methylation level of each site, the data were analyzed using modeling individual Illumina beta values using a generalized linear model (glm) with cell type (LCLs or PBCs), age and sex as the independent variables. *P* values and the difference in methylation level for each cell type were obtained. The statistical power to detect methylation differences of 0.25 and 0.5 between 192 PBCs and 92 LCLs was estimated to be 50.2 and 97.5%, respectively at a significance level of *P* = 0.05 using G*Power 3.1 software [10]. A principal component analysis (PCA) was performed using the beta values for the 400,240 sites, and the first and second principal component scores for each sample were plotted. The regression analysis was performed using the chronological age of the subjects and the beta values of cg06639320 and cg16867657 for *FHL2* and *ELOVL2*, respectively, with adjustments for sex. These analyses were performed using R (release 2.15.2).

## Results

### Comparison of global DNA methylation profiles between LCLs and PBCs

**Fig. 1:**
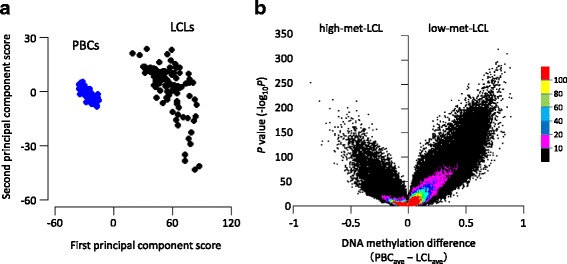
Global difference in the DNA methylation level between the LCLs and PBCs. **a** Principal component analysis (PCA) plot. PCA was performed using the methylation level of the 400,240 sites on autosomes. The LCL and PBC samples are shown in *black* and *blue dots*, respectively. **b** Volcano plot with the difference of the average of DNA methylation level on the *x*-axis and the *P* value (−log_10_ *P*) obtained via glm analysis on the *y*-axis. Each *color* shows the dot density (100 < *n*, 80 < *n* ≤ 100, 60 < *n* ≤ 80, 40 < *n* ≤ 60, 20 < *n* ≤ 40, 10 < *n* ≤ 20 and *n* ≤ 10 per unit area (0.002 × 1 for *x* and *y*-axis, respectively) in *red*, *yellow*, *green*, *sky blue*, *blue*, *pink*, and *black*, respectively)

To assess the global difference of DNA methylation levels between LCLs and PBCs, we performed a PCA using the methylation data of 400,240 sites on autosomes obtained using the 450 K methylation array. As shown in Fig. 1a, the LCL and PBC groups were clearly distinguished by their first principal component score. Additionally, the PBC samples were distributed within a narrow range, whereas the LCL samples showed a relatively wide range in the second principal component score. These results suggest that there is a global difference in DNA methylation levels between these cell types and that the levels are more diverse in LCLs than in PBCs.

We then examined the difference in methylation level for each site using a glm adjusted for age and sex. As shown in the volcano plot in Fig. 1b, the sites showing lower levels in LCL than in PBC were predominant (low-met-LCL group). The 138,871 sites (34.7% of the total) showed − log_10_(*P* value) > 10; among these sites, 85.1% were in the low-met-LCL group. This inclination was observed in each autosome (Additional file 1: Figure S1). Therefore, it was suggested that the main difference in DNA methylation between LCLs and PBCs was hypomethylation in the LCLs and that the change in methylation levels occurred globally in the autosomes.

### Hypomethylation observed in the LCLs occurs at sites outside the GpG island

**Fig. 2:**
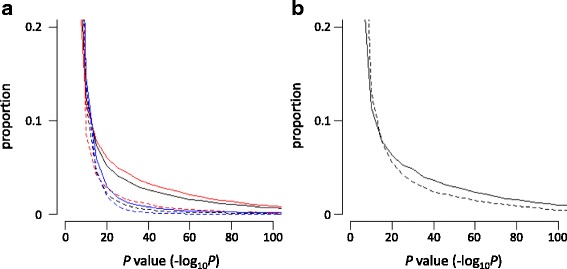
Distribution of the differences in methylation levels between LCLs and PBLs in terms of CGI. **a** The proportion of *P* values obtained from non-CGI and CGI sites in all samples (*black solid* and *dashed lines*, respectively), non-CGI and CGI sites in the low-met-LCL group (*red solid* and *dashed lines*, respectively), and non-CGI and CGI sites in the high-met-LCL group (*blue solid* and *dashed lines*, respectively) are indicated. **b** The proportion of *P* values obtained from the non-shore and shore sites (*solid* and *dashed lines*, respectively) in the non-CGI sites of the low-met-LCL group are indicated

We next assessed the distribution of the difference in methylation levels between LCLs and PBLs in terms of the location of the site (inside or outside the CpG island) (named CGI-site or non-CGI-site). As shown in Fig. 2a, the distribution of difference was dissimilar between them; the proportion of the sites showing a low *P* value was larger in the non-CGI-site group (black solid line) than in the CGI-site group (black dashed line). This trend was apparent in the low-met-LCL group (compare the red solid and dashed lines), whereas a dissimilarity of distribution was not observed in the high-met-LCL group (compare the blue solid and dashed lines). These results prompted us to further classify the non-CGI-sites into shore or non-shore groups because the CGI shores were suggested to contribute to tissue-specific DNA methylation [11, 12]. However, we did not find significant differences in the distribution between the shore and non-shore group of the low-met-LCL (Fig. 2b). Taken together, these results suggested that the majority of hypomethylation observed in the LCLs occurred at sites outside the CGIs regardless of shores.

### Comparison of the difference in DNA methylation levels observed among LCLs and PBCs in terms of distance from the transcription start site

**Fig. 3:**
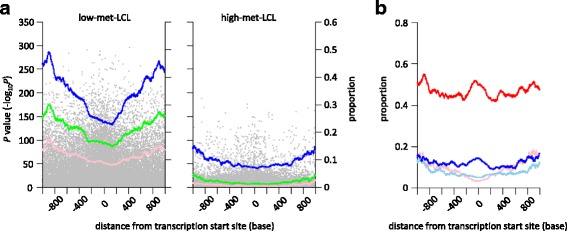
Distribution of the differences in methylation levels between LCLs and PBLs in terms of TSS. **a** *P* values were plotted against the distance from the nearest TSS (*gray dots*). The proportion of the sites with *P* values (−log_10_ *P*) greater than 10 (*blue dots*), 25 (*green dots*), and 50 (*pink dots*) in a window size of ±50 bases were plotted. **b** The proportion of the sites with *P* values (−log_10_ *P*) greater than 10 obtained from non-CGI and CGI sites in the low-met-LCL group (*red* and *pink dots*, respectively), and from non-CGI and CGI sites in the high-met-LCL group (*blue* and *sky blue dots*, respectively) in a window size of ±50 bases were plotted against TSS

We further examined the relationship between the distance from the TSS and the difference in DNA methylation levels observed among LCLs and PBCs. We plotted − log_10_(*P* value) for each site against the distance from the nearest TSS (shown in gray dots in Fig. 3a) and indicated a proportion of the site showing − log_10_(*P* value) > 10, 25, and 50 in blue, green, and pink dots, respectively (Fig. 3a). The proportion was calculated by dividing the number of the sites meeting the *P* value criteria by the total number of sites within ±50 bases of window size. We found that the proportion of significantly different sites was lower near the TSS. For instance, approximately 25% of the sites near the TSS showed − log_10_(*P* value) > 10, whereas this proportion increased to approximately 45% for the sites located approximately ±1000 bases from the TSS in the low-met-LCL group (blue dots, left panel of Fig. 3a). This trend was also observed even in the lower *P* value threshold group (green and pink dots) and in the high-met-LCL group (right panel of Fig. 3a). We then analyzed the sites showing − log_10_(*P*-value) > 10 separately for CGI- and non-CGI-site groups. As shown in Fig. 3b, the proportion of non-CGI-sites near the TSS was high in both the low- and high-met-LCL groups (red and blue dots, respectively, Fig. 3b). However, the lowest proportion was observed near the TSS in the case of CGI-sites (pink and sky blue dots for low- and high-met-LCL groups, respectively, Fig. 3b). These results suggested that the low CpG promoter would show a more significant difference in DNA methylation levels than the high CpG promoter.

### The methylation sites located in low CpG promoters could be sensitive to demethylation in LCLs

**Fig. 4:**
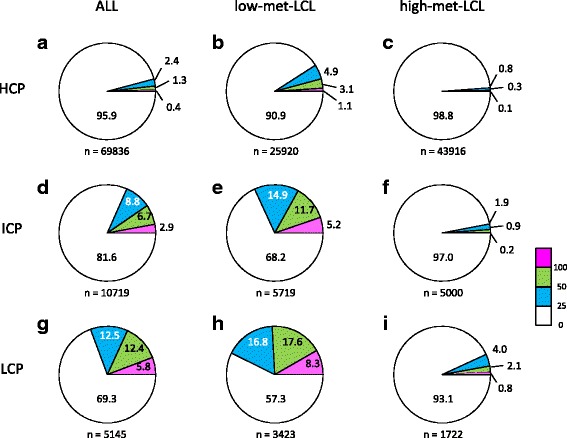
Difference in methylation levels between LCLs and PBLs in terms of promoter type. The proportion of the sites with *P* values (−log_10_ *P*) ≤ 25, 25 ~ 50, 50 ~ 100, and ≥ 100 are indicated in *white*, *blue*, *green*, and *red*, respectively. The results obtained from the HCP, ICP, and LCP sites in all samples (**a**, **d**, and **g**, respectively) in the low-met-LCL group (**b**, **e**, and **h**, respectively) and in the high-met-LCL (**c**, **f**, and **i**, respectively) are shown

To assess whether the promoter type affects the difference in DNA methylation levels between LCLs and PBCs, the methylation sites located in HCP, LCP and ICP were extracted based on the data set of Mikkelsen et al. [9] (69,836, 10,719, and 5,145, in HCP, ICP, and LCP, respectively), and analyzed the distribution of − log_10_(*P* value) in all, low- and high-met-LCL groups (Fig. 4). It was shown that the proportion of differentially methylated sites was higher in the LCPs than the HCPs. In the LCPs, the proportion of the sites showing − log_10_(*P* value) > 25 was 30.7%, whereas that in HCPs was 4.1% in all sites (compare Fig. 4a, g). This was more pronounced in the low-met-LCL group (compare Fig. 4b, c, h, i). The sites located in ICPs showed intermediate values between HCPs and LCPs (Fig. 4d–f). These results suggested that the methylation sites located in low CpG promoters could be sensitive to demethylation in LCLs.

**Fig. 5:**
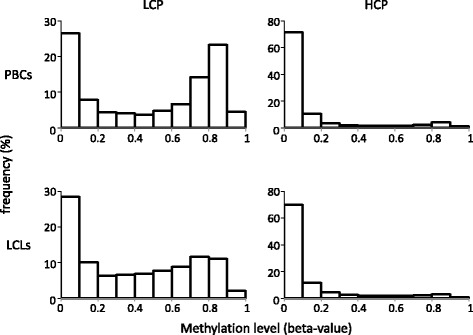
Distribution of the methylation levels of the sites in the LCPs and HCPs. The frequency in the LCLs and PBCs are shown separately

To further assess promoter type differences, we compared the HCPs and LCPs methylation level profiles. As shown in Fig. 5, nearly half of the sites in LCPs showed more than 0.6 methylation levels, whereas almost all sites in HCPs were hypomethylated in PBCs. Additionally, it was observed that the methylation levels of highly methylated sites of the LCPs decreased in the LCLs. Therefore, we concluded that highly methylated sites of LCPs caused the difference in DNA methylation levels observed between HCPs and LCPs, especially in the low-met-LCL group.

### Comparison between LCLs and PBCs regarding the association between ageing-related CpG sites and chronological age

**Fig. 6:**
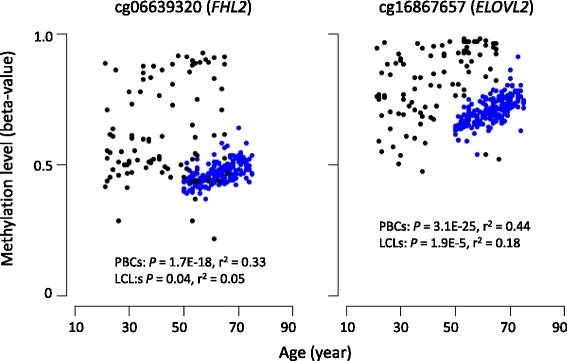
Regression analyses of the methylation levels and chronological age at the *FHL2* and *ELOVL2* loci. The methylation levels in the LCLs (*black dots*) and PBCs (*blue dots*) were plotted against the age of the donors at the time of providing the specimens. The *P* values and *r* ^2^ were obtained by correcting for sex

Using DNA obtained from PBCs, it has been reported that the methylation levels of several CpG sites are associated with chronological age. However, it remains unclear whether LCLs should be utilized for studies on epigenetic ageing biomarkers. To address this issue, we performed a regression analysis for chronological age and known ageing-related CpG sites located in *FHL2* and *ELOVL2* [13, 14]. *FHL2* encodes a member of the four-and-a-half-LIM-only protein family which is suggested to have a role in the assembly of extracellular membranes and in transformation of normal myoblasts to rhabdomyosarcoma cells (OMIN 602633). *ELOVL2* encodes an enzyme which catalyzes the first and rate-limiting reaction of the long-chain fatty acids elongation cycle (OMIM 611814). As shown in Fig. 6a, the methylation level of the PBCs was highly correlated with chronological age (blue dots, *P* = 1.7E-18 and *r* ^2^ = 0.33 for *FHL2*, *P* = 3.1E-25 and *r* ^2^ = 0.44 for *ELOVL2*). In contrast, the methylation level of the LCLs was varied and the association was weak (black dots, *P* = 0.04 and *r* ^2^ = 0.05 for *FHL2*, *P* = 1.9E-5 and *r* ^2^ = 0.18 for *ELOVL2*). Therefore, these results suggest that DNA obtained from LCLs may not always be an alternative to DNA from PBCs.

## Discussion

In this study, we used a 450 K methylation array to investigate the methylation differences between LCLs and PBCs, which are commonly used in genetic epidemiological studies. In all genomes, the majority of the sites in the LCLs showed lower methylation levels than those of the PBCs, and these sites were primarily located in non-CGI regions. Additionally, we found that differentially methylated sites were predominantly located in the LCP region.

Although a relatively small sample number and number of methylation sites were analyzed, previous studies showed that methylation status in LCLs is different from that of PBCs and that the methylation level in LCLs is lower than that of PBCs in the majority of sites [15–20]. Because a large number of samples and more sites were examined, we could investigate the differences in methylation levels between LCLs and PBCs in terms of CGI location, distance from TSS and promoter type as characterized by CG density. We found that a fraction showing a significant difference in methylation level between the LCLs and PBCs was observed near the TSS in the non-CGI sites but not in the CGI sites. This result suggests that the difference in the methylation level of these cell types would be high in the genes in which the promoter shows a low GC content.

We found that significantly different methylation sites were predominant in LCPs but not in HCPs. It has been demonstrated that LCPs are generally associated with tissue-specific genes, whereas HCPs are associated with two classes of genes, including ubiquitous “housekeeping” genes and highly regulated “key developmental” genes [9, 21, 22]. Therefore, our results suggest that the methylation sites located in promoters classified as LCP could have a functional role in distinguishing between LCLs and PBCs by regulating the corresponding gene expression.

The epigenome-wide association studies using human population samples to identify the disease risk loci and epigenomes that are affected by intrinsic or extrinsic factors, such as ageing and smoking, have been progressing [13, 14, 23, 24]. We evaluated the differences in association strength between well-known ageing methylation sites and the chronological age of the samples between LCLs and PBCs and found that the correlation was more significant in PBCs than LCLs. This was due to a larger variance of methylation levels in LCLs than in PBCs. In addition to the differences in cell type, artificial experimental processes, including in vitro culture, culture period and culture freezing, and thawing could cause the large variances in data observed in the LCLs. Therefore, we concluded that DNA obtained from LCLs may not always be a proxy for DNA from PBCs in studies of epigenome-wide analysis attempting to elucidate the role of epigenetic change in disease risks.

## Conclusion

There is a global difference in DNA methylation levels between LCLs and PBCs, and the main difference was hypomethylation in the LCLs. The methylation levels of highly methylated sites of the low-CpG-density promoters in PBCs decreased in the LCLs, suggesting that the methylation sites located in low-CpG-density promoters could be sensitive to demethylation in LCLs. The correlation between well-known ageing methylation sites and the chronological age of the samples was more significant in PBCs than LCLs, indicating that despite being generated from a single cell type, LCLs may not always be a proxy for DNA from PBCs in studies of epigenome-wide analysis attempting to elucidate the role of epigenetic change in disease risks.

## Additional file

Additional file 1: Figure S1. Volcano plot for each autosome with the difference of the average of the DNA methylation levels on the x-axis and the *P* value (−log_10_ *P*) obtained via glm analysis on the y-axis. Each color shows the dot density (100 < *n*, 80 < *n* ≤ 100, 60 < *n* ≤ 80, 40 < *n* ≤ 60, 20 < *n* ≤ 40, 10 < *n* ≤ 20, and *n* ≤ 10 per unit area (0.002 × 1 for the *x*-axis and *y*-axis, respectively) in red, yellow, green, sky blue, blue, pink and black, respectively). (PDF 295 kb)

## Abbreviations

CGI: CpG Island
ELOVL2: Elongation of very long chain fatty acids protein 2
FHL2: Four and a half LIM domains 2
Glm: Generalized linear model
HCP: High-CpG-density promoter
ICP: Intermediate-CpG-density promoter
LCL: Lymphoblastoid cell line
LCP: Low-CpG-density promoter
PBC: Peripheral blood cell
PCA: Principal component analysis
TSS: Transcription start site
