# Supplementary material for: Genome-wide DNA methylation analysis reveals hypomethylation in the low-CpG promoter regions in lymphoblastoid cell lines
Source: Hum Genomics. 2017 May 12;11:8. doi: 10.1186/s40246-017-0106-6 (PMC5429538; doi:10.1186/s40246-017-0106-6)
Supplement: Additional file 1: Figure S1. — Volcano plot for each autosome with the difference of the average of the DNA methylation levels on the x-axis and the P value (−log10 P) obtained via glm analysis on the y-axis. Each color shows the dot density (100 < n, 80 < n ≤ 100, 60 < n ≤ 80, 40 < n ≤ 60, 20 < n ≤ 40, 10 < n ≤ 20, and n ≤ 10 per unit area (0.002 × 1 for the x-axis and y-axis, respectively) in red, yellow, green, sky blue, blue, pink and black, respectively). (PDF 295 kb) [file 40246_2017_106_MOESM1_ESM.pdf]

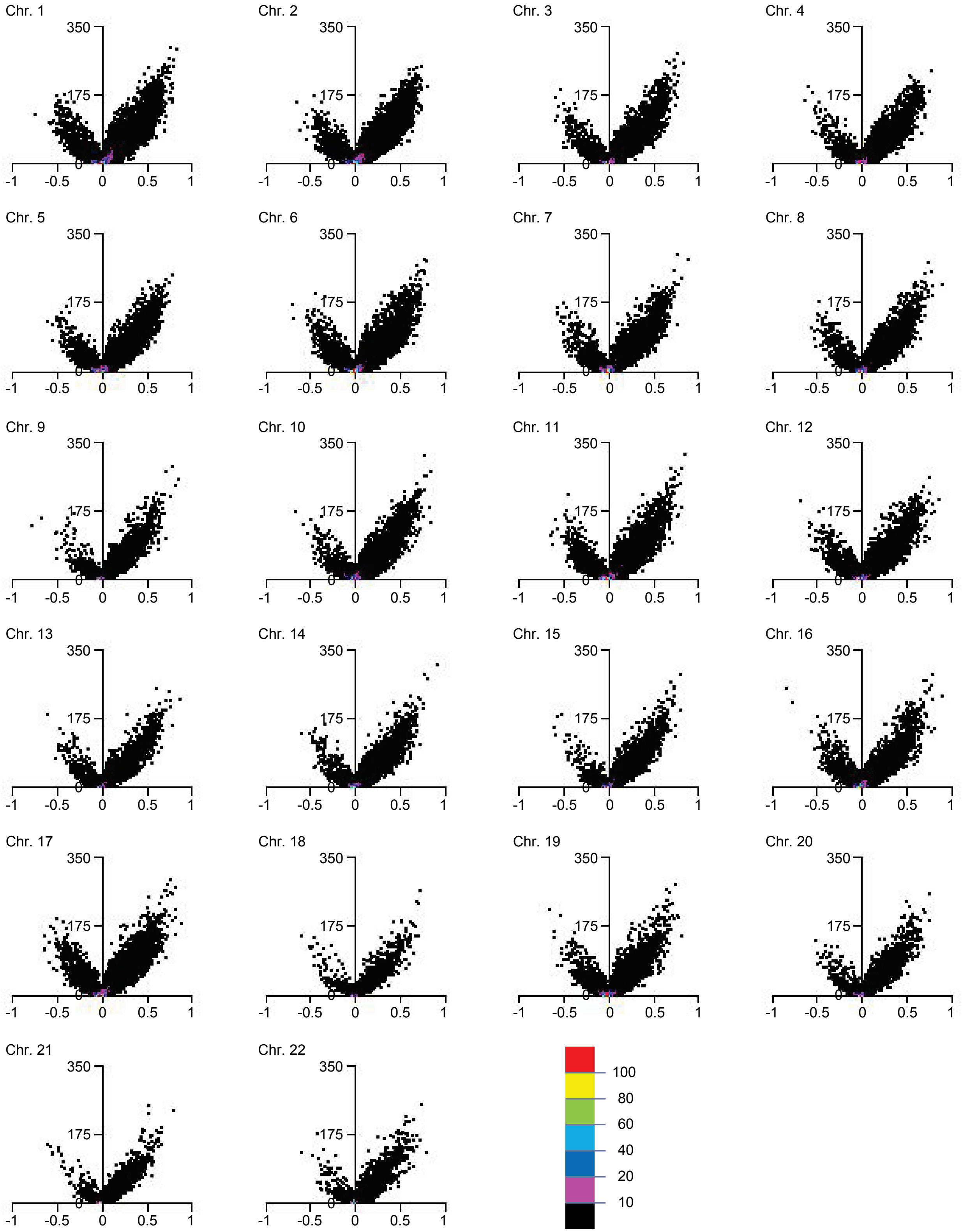

Supplementary Figure 1

Volcano plot for each autosome with the difference of average of DNA methylation level in x-axis and the *P* value ($-\log_{10}P$) obtained by glm analysis in y-axis. Each color shows dot density (100 < n, 80 < n ≤ 100, 60 < n ≤ 80, 40 < n ≤ 60, 20 < n ≤ 40, 10 < n ≤ 20 and n ≤ 10 per unit area (0.002 × 1 for x and y-axis, respectively) are depicted by red, yellow, green, sky blue, blue, pink and black, respectively).
